# Sorting of Odor Dilutions Is a Meaningful Addition to Assessments of Olfactory Function as Suggested by Machine-Learning-Based Analyses

**DOI:** 10.3390/jcm11144012

**Published:** 2022-07-11

**Authors:** Jörn Lötsch, Anne Huster, Thomas Hummel

**Affiliations:** 1 Institute of Clinical Pharmacology, Goethe-University, Theodor-Stern-Kai 7, 60590 Frankfurt am Main, Germany; 2 Fraunhofer Institute for Translational Medicine and Pharmacology ITMP, Theodor-Stern-Kai 7, 60596 Frankfurt am Main, Germany; 3 Smell & Taste Clinic, Department of Otorhinolaryngology, TU Dresden, Fetscherstrasse 74, 01307 Dresden, Germany; anne.huster@tu-dresden.de (A.H.); thomas.hummel@tu-dresden.de (T.H.)

**Keywords:** olfaction, olfactory testing, patients, data science, machine learning

## Abstract

Background: The categorization of individuals as normosmic, hyposmic, or anosmic from test results of odor threshold, discrimination, and identification may provide a limited view of the sense of smell. The purpose of this study was to expand the clinical diagnostic repertoire by including additional tests. Methods: A random cohort of n = 135 individuals (83 women and 52 men, aged 21 to 94 years) was tested for odor threshold, discrimination, and identification, plus a distance test, in which the odor of peanut butter is perceived, a sorting task of odor dilutions for phenylethyl alcohol and eugenol, a discrimination test for odorant enantiomers, a lateralization test with eucalyptol, a threshold assessment after 10 min of exposure to phenylethyl alcohol, and a questionnaire on the importance of olfaction. Unsupervised methods were used to detect structure in the olfaction-related data, followed by supervised feature selection methods from statistics and machine learning to identify relevant variables. Results: The structure in the olfaction-related data divided the cohort into two distinct clusters with n = 80 and 55 subjects. Odor threshold, discrimination, and identification did not play a relevant role for cluster assignment, which, on the other hand, depended on performance in the two odor dilution sorting tasks, from which cluster assignment was possible with a median 100-fold cross-validated balanced accuracy of 77–88%. Conclusions: The addition of an odor sorting task with the two proposed odor dilutions to the odor test battery expands the phenotype of olfaction and fits seamlessly into the sensory focus of standard test batteries.

## 1. Introduction

Clinical tests or test batteries for the sense of smell typically involve the evaluation of the olfactory threshold for an odorant, the discrimination of different odorants, and the identification of culturally known odors. Today’s olfactory test batteries include all of these components [1,2] or a subset of these tests [3,4,5,6]. Efforts have even been made to minimize the tests used, to the point of inferring the olfactory function from the ability to recognize a single odor or a few odors only, e.g., five odors or less [7,8,9]. The reduction of olfactory tests to a few odor-identification tasks reduces the burden of olfactory diagnostics and therefore facilitates their inclusion in routine clinical assessments and screening tests of larger populations. However, such simple tests also reduce the resolution of olfactory function assessment [10] and generally only allow assignment to a categorical diagnosis of anosmia or normosmia [7,8,11]. More comprehensive tests allow a more detailed scoring of olfactory function. For example, for the Sniffin’ Sticks test, a score change of 5.5 points has been identified as the threshold above which changes in olfactory function are subjectively perceived, i.e., the minimal clinically significant difference [12]. Such a resolution is not available with short screening tests. With increasing attempts to treat olfactory dysfunction through pharmacological approaches or sensory training [13], the three test components may still not suffice to capture all aspects of olfactory changes. The exploration of informative extensions of standard clinical tests of olfactory function therefore seems warranted.

The present study was designed to evaluate possible additions to a standard odor test battery that assesses the olfactory threshold, odor discrimination, and odor identification [1,2]. This included additional sensory tests such as sorting tasks for the dilution series of odorants, the evaluation of the olfactory threshold after prolonged exposure to an odor to assess adaptation or habituation of the sense of smell or psychosocial aspects such as the subjective importance of the sense of smell in everyday life. It was hypothesized that informative sensory olfactory phenotypes are likely to be more complex than threshold, identification, and discrimination scores allow to conclude, with no specific preference for any of the additional tests assessed. Therefore, this exploratory study was analyzed using a data-driven approach with initial unsupervised assessments of structures in the dataset that are indicative of odor-related phenotypes that can subsequently be interpreted from the clinical perspective of an expert in human olfaction.

## 2. Methods

### 2.1. Patients and Study Design

The prospective cohort study was conducted in accordance with the Declaration of Helsinki on Biomedical Studies Involving Human Subjects. It was approved by the Ethics committee at the Dresden University Hospital (approval number EK278082019). All participants gave informed written consent. Participants were n = 135 patients, 83 women and 52 men, aged between 21 and 94 years (mean ± standard deviation, SD: 30.5 ± 11.6 years) and with a body mass index (BMI) between 16.3 and 41.2 kg/m^2^ (means SD: 23.1 ± 4 kg/m^2^). They were recruited either through flyers or through the Smell and Taste Clinic at the Department of Otorhinolaryngology of the TU Dresden. Inclusion criteria were age 18 years and older and subjectively normal olfactory function in healthy controls or the presence of olfactory disorder in patients with olfactory loss. Exclusion criteria were pregnancy, lactation, smoking (>5 cigarettes per week), acute nasal inflammation, neurodegenerative disorder (e.g., Parkinson’s disease), or other diseases that frequently are associated with olfactory loss. Measurements took place between October 2019 and June 2021.

### 2.2. Acquisition of Olfaction Related Variables

Measurements were taken in the following sequence (abbreviations in bold in brackets): Assessment of olfactory threshold, odor discrimination and identification performance (**Sniffin’ Sticks: olfthresh, olfdis, olfident)**, a distance test with peanut butter (**peanut**), ordering of odor intensities for phenylethyl alcohol [PEA] and eugenol [EUG] (**PEA order, EUG order**), a discrimination test for odor enantiomers (**enantiomer**), lateralization test using eucalyptol (**lateral**), the threshold test after odor exposure using PEA-filled nose clips (**adaptation**), and finally, a questionnaire on the importance of olfaction (**Importance**). The entire test battery including breaks took approximately 90 min. The measurements took place in well-ventilated, relatively quiet rooms of the same experimenter (AH). The participants received moderate financial compensation. The tests are described in more detail below; the names of the analyzed variables are described in the legend of Table 1.

#### 2.2.1. Assessment of Olfactory Threshold, Odor Discrimination, and Identification Performance

The functional performance of subjects’ sense of smell was assessed by evaluating olfactory threshold and performance in odor discrimination and identification tasks. Today’s test batteries include all or a subset of the components [3,4,5,6], and in the present study, the “Sniffin’ Sticks” test battery (Burghart Instruments, Wedel, Germany) was used, in which odors are presented in felt-tip pens approximately 14 cm long with a 1.3 cm inside diameter at 20 s intervals [1,2]. The olfactory threshold (**olfthresh**) was determined for phenylethanol dissolved in propylene glycol in 16 dilution steps in a geometric series starting with a 4% odor solution. Triples of pens were presented, one of which contained the diluted odor, while the other two were blanks. Subjects had to identify the odorous pen in a three-alternative forced-choice paradigm (3-AFC). Using a staircase paradigm, two consecutive correct identifications triggered the transition to the next higher dilution, while one incorrect identification triggered the return to the next lower dilution. After seven turning points, the threshold was calculated as the arithmetic mean of the dilution steps of the last four turning points. Odor discrimination (**olfdis**) was also assessed in a 3-AFC design using 16 triplets with two pens with the same odorant and one pen with a different odor, with the task being to identify the pen with the different odor. Odor identification (**olfident**) was assessed in a 6-AFC design in which subjects had to name 16 odors from 6 alternatives given with each odorant. This expansion of the standard version employs a 4-multiple forced-choice paradigm as proposed in [14]. The final TDI score was the sum of the scores for the threshold, discrimination, and identification subtests with a range between 1 and 48 points. Using the TDI score, olfactory function diagnosis was determined as either functional anosmia (further referred to as “anosmia”; score < 16.5), hyposmia (16.5–30.5), or normosmia (>30.5) [15].

#### 2.2.2. Distance Test with Peanut Butter

The “**Peanut test**” is based on the lateralized measurement of the distance at which an odor is perceived. It was first introduced by Stamps et al. [16], similarly to the alcohol sniff test first published by Davidson and Murphy [17]. The idea of the test is to assess the ability of individuals to perceive odors from a distance, which also reflects olfactory sensitivity. To this end, an odor source (brown glass jars of 50 mL volume, round opening, opening diameter 32 mm, filled with 14g peanut butter: “American Creamy Peanut Butter”, CMC The Food Company GmbH, Mühlheim, Germany) is moved towards the nostril, beginning at a distance of 30 cm [16]. In the sitting participant, the odor source is slowly moved upwards along a ruler parallel to the body axis, in 1 cm steps per exhalation. During the measurements, participants closed their eyes and gently closed one nostril with their finger. Care was taken not to deform the nasal anatomy on the contralateral side. Participants continue to breathe calmly and evenly with their mouths closed. They signaled as soon as they smelled the peanut butter. At that moment, the experimenter read the distance between the nostril and the opening of the jar [16]. This process was repeated three times per nostril. The mean value was used as an estimate of the distance. Intervals of approximately 90 s were kept between these trials. The side of the first measurement was randomized across participants. The tested nostril changed with each measurement.

#### 2.2.3. Ordering of Odor Intensities for Phenylethyl Alcohol and Eugenol

**Figure 1:**
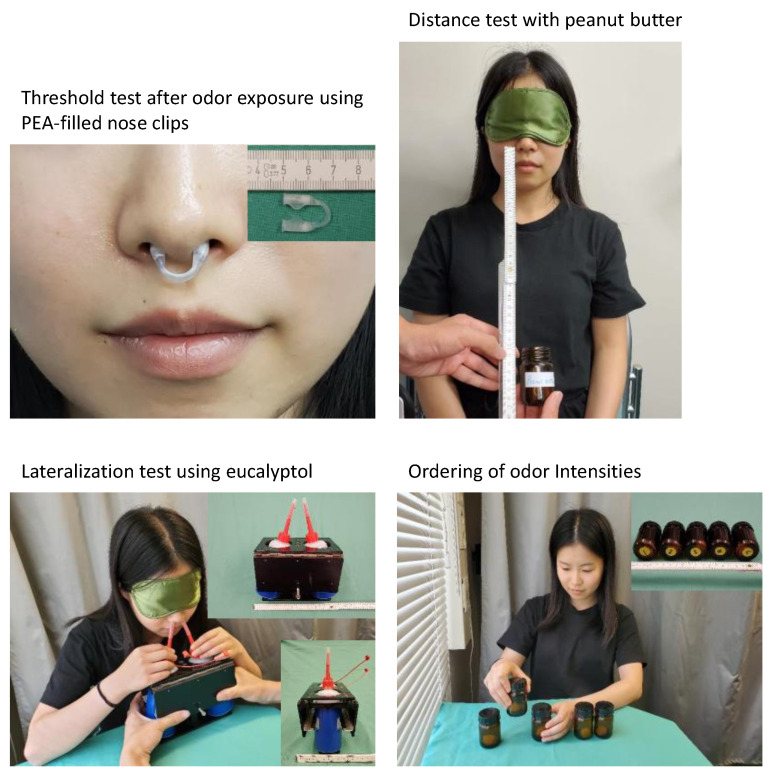
Photographs showing details of the administration of olfactory tasks. **Top left**–nasal clip (see also insert) filled with phenylethylalcohol to provide continuous olfactory background stimulation. **Top right**—distance test with an opened jar with peanut butter slowly moved upwards towards the nose of the blinded participant with a meter to measure the distance from the nares. **Bottom left**—lateralization task using a hand-held squeezing device (see also inserts), which allows to administer the same amount of air to the left and right nostrils of the blinded participant with one bottle containing eucalyptus. **Bottom right**—odor-sorting task with the participant arranging odor-containing bottles according to the different odor intensities with odor concentrations indicated at the bottom of the bottle (see also insert).

The order tasks for phenylethyl alcohol (PEA) and eugenol (EUG) (“**PEA order test**”, “**EUG order test**”, respectively) were meant to address olfactory skills that are needed in the daily functioning of the sense of smell (Figure 1). This task involved several skills, e.g., odor memory, sensitivity to odorous sensation, and olfactory adaptation. A similar test had been proposed earlier [18]. Five dilutions each were prepared for the fragrances PEA and EUG, starting from a 1% concentration using the solvent propylene glycol, in dilution steps of 1:2 (2PEA, order number 77861; EUG, order number W246700; propylene glycol, order number W294004; Sigma-Aldrich, Taufkirchen, Germany). Odors were presented in brown glass jars of 50 mL volume, with a round opening of 32 mm diameter. The jars had screw-on caps. Participants were not limited in time to organize the 5 concentrations in ascending order. As soon as an order had been established, the participant notified the experimenter who noted down the time required for the task. The score was the absolute difference between the assigned rank and the actual rank, e.g., if a subject placed the third dilution in the second position, the difference was 1, or if instead of the last position, the most concentrated odor was placed in position 1, the difference was 4. Thus, a higher score indicated poorer performance, whereas a perfect sorting yielded zero difference.

#### 2.2.4. Discrimination Test for Odor Enantiomers

Like the odor discrimination task of the Sniffin Sticks, the ***enantiomer test*** was meant to be a challenge for participants with a good sense of smell. To this end, the test was based on pairs of enantiomers with similar smells presented within 3-alternative forced-choice tasks. The following pairs were used: S-(−)-limonene/R-(+)-limonene, (−)-fenchone/ (+)-fenchone, L-carvone/ R-(−)-carvone, and (R)-(−)-2-butanol/ (L)-(+)-2-butanol with R/(+)-enantiomers presented twice (substance: order number: dilution in propylene glycol; S-(−)-limonene: 62130: 17%; R-(+)-limonene: 62120: 100%; (−) fenchone: 46200: 100%; (+)-fenchone: 46210: 100%; L carvone: W224908: 17%; R-(−)-carvone: 124931: 100%; (R)-(−)-2-butanol: 236691: 100%; (L)-(+)-2-butanol: 237698: 100%; all odors from Sigma-Aldrich). The task was performed similarly to the odor discrimination task presented with Sniffin’ Sticks (see above) with the exception that odors were presented in glass jars of 50 mL volume, with a round opening of 32 mm diameter. Prior to testing, odor intensities had been matched by a group of 16 healthy participants in order to establish isointense stimuli (12 women and 4 men, age 24-55 years, mean age 27.8 years, SD 7.5 years). Based on their ratings, L-carvone and S-limonene were diluted at 1:5.

#### 2.2.5. Lateralization Test Using Eucalyptol

The **lateralization test** is used to assess nasal trigeminal sensitivity, which has been shown to be closely associated with olfactory function [19,20]. To this end, lateralization abilities were quantified with the use of a mechanically operated stimulation device. The trigeminal stimulus eucalyptol was presented (15 mL of 50% eucalyptol solved in propylene glycol; eucalyptol order numberC80601; Sigma-Aldrich, Taufkirchen, Germany) to either nostril, while the other received air from a bottle filled with 15 mL of propylene glycol (compare [21,22]). The compressible polypropylene bottles had a volume of 250 mL each. Stimuli with a volume of 15 mL each were released from the left and the right bottles during each stimulation. The bottles had a spout, which was fitted with disposable, soft silicon tubing (inner diameter 5 mm) so that possible irritation at the nares was minimized. For each stimulus presentation, subjects were instructed to hold onto the outlets so that the tubing reached inside the nares for approximately 1 cm beyond the nasal valve area. Following each stimulus presentation, participants raised their left or right hand to indicate the side of stimulation. First, two test runs were carried out so that participants could familiarize themselves with the procedure. A total of 40 stimuli (20 at each side of the nose) were then applied in pseudo-randomized order to the blindfolded participants, with an interval of approximately 30 s between trials. The score was the sum of correct lateralizations [23].

#### 2.2.6. Threshold Test after Odor Exposure Using PEA-Filled Nose Clips

The **adaptation test** was meant to provide a challenge for healthy individuals with a good sense of smell. Because adaptation is typically seen after exposure to odors [24,25], we measured the degree to which the PEA odor threshold is affected by previous exposure to PEA. To this end, participants received a nose clip (Aspuraclip, Schönefeld, Germany). The clips were filled with a total of 0.3 mL PEA. They were made of an elastic silicone tube, in a horseshoe-like shape (18 mm length of legs of the U-shape, 3 mm diameter of tubing), which allowed insertion of the clip into the left and right nostril, attached to the nasal septum. The clip was worn for 10 min during which participants were breathing calmly and evenly, with their mouths closed. Wearing the clips did not cause any discomfort. Following removal of the clip, the PEA odor threshold was determined using the Sniffin’ Sticks test kit as described above.

#### 2.2.7. Questionnaire—Importance of Olfaction

The questionnaire on the importance of olfaction (***Importance test***) is comprised of 18 statements on the importance of smells in everyday situations (Croy et al., 2010). Each question is scored with a four-point self-assessment scale, ranging from “completely agree”, corresponding to a numerical value of three, to “do not agree at all”, corresponding to a numerical value of zero. Six of the statements are combined into scores for three subcategories. The elements of the scale “association” refer to the ability of scents to evoke emotions or memories. The “application” scale relates to the conscious use of the olfactory system in everyday situations. The “consequence” scale reflects the extent to which individual decisions are influenced by olfactory information. In addition, the questionnaire contains two further statements, which are recorded under the term aggravation or lie score. They provide conclusions about the tendency to overestimate—these were disregarded for the present study. Based on the 6 statements in each of the 3 main categories, a maximum of 18 points could be received. In general, higher scores imply greater individual importance of olfaction and vice versa [26].

### 2.3. Data Analysis

Data analysis was performed using unsupervised methods to detect correlation and cluster structures in the olfaction-related data, followed by a supervised analysis of differences between detected subgroups. The necessary programming work was performed in the Python language [27] using Python version 3.8.12, which is available free of charge at https://www.python.org (accessed on 28 January 2022). Experiments were performed in the Anaconda data science environment (Anaconda Inc., Austin, TX, USA), freely available at https://www.anaconda.com, accessed on 28 January 2022), on an AMD Ryzen Threadripper 3970X (Advanced Micro Devices, Inc., Santa Clara, CA, USA) computer, running on Ubuntu Linux 20.04.4 LTS (Canonical, London, UK)). The main packages used for the data analysis were the numerical Python package “numpy” (https://numpy.org, accessed on 28 January 2022 [28]), “pandas” (https://pandas.pydata.org, accessed on 28 January 2022 [29,30]), fundamental algorithms for scientific computing in Python “SciPy” (https://scipy.org [31]), and “scikit-learn” (https://scikit-learn.org/stable/, accessed on 28 January 2022 [32]).

#### 2.3.1. Data Preprocessing

Data preprocessing included examination of the distribution of the variables, including evaluation of possible transformations along Tukey’s ladder of powers [33,34], supported by visualizing the data using quantile–quantile plots and assessing the normal distribution using D’Agostino and Pearson tests [35,36] implemented in the “SciPy” Python package. This suggested logarithmic transformation of olfactory thresholds, consistent with the geometric scaling of odorant dilutions applied during their assessment and of the distances from odor to nostril measured in the Peanut butter test. Reciprocal square root transformation was indicated for BMI. Imputation of missing values was performed for the olfaction-related variables using random forests [37,38] implemented in the Python package “miceforest” (https://pypi.org/project/miceforest/, accessed on 28 January 2022). Only variables or cases with less than 20% missing values were retained.

#### 2.3.2. Unsupervised Analysis of Olfaction-Related Data

Following transformation and imputation of the variables, unsupervised analyses for identifying structures in the olfaction-related data comprised the calculation of Pearson’s product-moment correlation coefficients [39] and the projection of the z-standardized variables onto uncorrelated planes by means of principal components analysis (PCA) [40,41]. There, the number of relevant components to retain was selected via the Kaiser–Guttman criterion of eigenvalues > 1 [42,43]. The importance of the relevant olfaction-related variables for each principal component was estimated from the loadings of each variable on the PCs weighted by the contribution of the PCs to the explanation of the total variance. Therefore, the dot product of the original (z-transformed) variables and the obtained principal components (PCs) was calculated, and the z-transformed resulting matrix was multiplied by the proportion of variance explained by each PC.

The row sums over the relevant PCs for each variable yielded the variables’ importance. To access the most relevant variables for the PCA projection, this importance measure was submitted to compute ABC analysis [44], an item categorization technique adopted from economic sciences that aims to divide a set of positive numerical data into three disjoint subsets labeled “A”, “B”, and “C”. Set “A” should contain the “important few” elements, i.e., the elements that make it possible to obtain maximum return with minimum effort [45]. The Python code “ABCanalysis” for this method is freely available at https://github.com/JornLotsch/ABCanalysis (accessed on 28 January 2022).

Cluster structures in the olfaction-related data were sought in the retained principal components (PCs) by k-means clustering [46,47] using the Euclidean distance between projected data points. The number of clusters was determined among k = 2,...,5 possible clusters using the mean silhouette width as the main criterion [48]. The quality and stability of the final clustering solution were evaluated by calculating the silhouette widths and the adjusted Rand index [49] in 20-fold cross-validation runs using bootstrap [50] resampling from the original data. Alternative clustering methods such as Ward’s agglomerative clustering [51] (see Supplementary Figures S1 and S2) or partitioning around medoids [52] provided lower silhouette values were therefore discarded.

#### 2.3.3. Supervised Analysis to Identify Olfactory Variables Relevant to the Structure of the Dataset

Supervised analyses for interpreting subgroups of subjects included statistical group comparisons using Mann–Whitney U [53] and χ^2^ tests [54]. Alpha correction for multiple testing was applied as suggested by Bonferroni [55]. In these analyses, age and BMI were included in addition to the olfaction-related variables used for clustering.

To further determine the relevant variables for the identified cluster structure, several feature selection methods were used [56]. These included (i) PCA-based variable importance described above and univariate feature selection methods implemented as (ii) calculation of effect sizes expressed as Cohen’s d [57] and (iii) F-value based selection as implemented in the “SelectKBest” method available in the “sklearn.feature_selection” module of scikit-learn. The number of k features to be selected as best was determined by a grid search of [1, ..., 18] variables in analyses based on the algorithms specified below. Further feature selection methods were based on the assignment of an importance measure to each variable following training of classification algorithms. Specifically, support vector machines (SVM [58]), implemented as “linearSVC”, and random forests [37,38] were selected as two commonly used classification algorithms of different types, i.e., class separation using hyperplanes in data projected to higher dimensions, or class separation using an ensemble of simple decision trees (for an overview of machine algorithms suitable for olfactory data, see [59]). In addition, logistic regression was included as a classical method for class assignment [60]. Following the training of the algorithms, the most relevant variables were selected using methods available in the “sklearn.feature_selection” module of scikit-learn, including (iv) “SelectFromModel” (SFM), which selects features based on importance weights in the trained algorithm, (v) recursive feature elimination (RFE), which selects features by recursively considering smaller and smaller feature sets and generating a feature ranking, and (vi) forward and backward sequential feature selection (SFS), which iteratively finds the best features by adding features to a set of initially zero and all features, respectively.

Algorithm-based feature selection was performed after 20% of the members of each cluster had been put aside as a validation sample, which was not further touched during algorithm training and feature selection. Subsequently, hyperparameter tuning was performed for the selected algorithms including using a 5-fold cross-validated grid search scenario as default in the “GridSearchCV” method of the “sklearn.model_selection” module of scikit-learn, during which the penalty measure was also chosen from the regularization methods (i) least absolute shrinkage and selection operator (LASSO) [61,62] or (ii) Ridge regression [63]. The feature selection methods were applied in a 100-fold cross-validation scenario provided with the “RepeatedStratifiedKFold” method from the “sklearn.model_selection” module of “scikit-learn”, setting the parameters “n_splits” = 5 and “n_repeats” = 20. From each cross-validation run, the selected variables were retained. The final feature sets for each selection method were determined by applying computed ABC analysis to the number by which each variable was selected in the set of 100 runs, from which the variables assigned to ABC category “A” were then retained. The tuning of the algorithms led to the selection of LASSO and Ridge regression as regularization methods for SVM and logistic regression, respectively. Other hyperparameter settings include d = 200 trees with a maximum depth of 10 decisions for random forests or the selection of the “newton-cg” solver for logistic regression.

Finally, a reduced feature set was determined from the sum count at which the variables had been selected in the 17 different approaches (Table 1), including PCA variable importance, effect size calculation via Cohen’s d, and the 15 machine-learning-based analyses that result from using five different selection methods and three different algorithms. The sum score across the selections was subjected to an ABC analysis. Finally, it was assessed whether this set of variables provided sufficient information for cluster separation in a sample not available during feature selection. The included algorithms were therefore trained with the full and reduced feature sets in a 100-fold cross-validation, using randomly selected subsets of 80% of the original training dataset for algorithm training, and applying the trained algorithms to random subsets comprising 80% of the validation dataset separated from the full original dataset before feature selection and classifier tuning. The balanced accuracy was used as the main parameter to evaluate the classification performance [64].

## 3. Results

**Figure 2:**
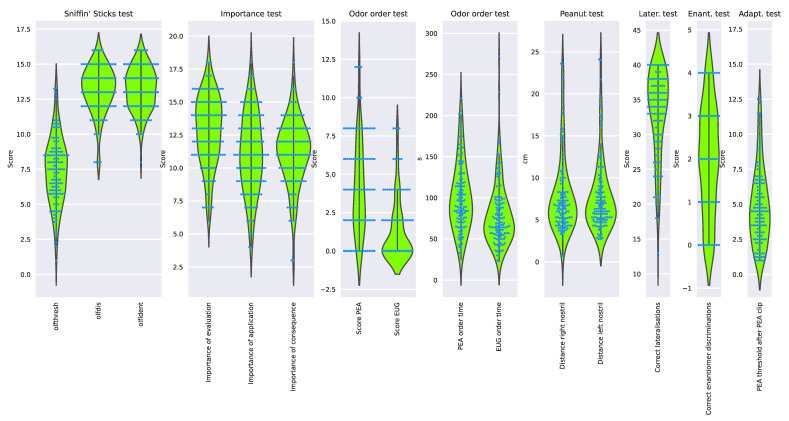
Raw non-transformed and non-imputed olfaction-related data acquired from n = 135 individuals. Single data points are plotted as dots on violin plots showing the probability density distribution of the variables, and in addition, boxplots provide basic descriptive statistics. Variable names, if not self-explaining: “olfthresh“ = olfactory threshold to phenyl ethyl alcohol (PEA), “olfdis” = score in the odor discrimination task, “olfident” = score in the odor identification task. Higher values indicate better olfactory performance. “log Distance right/left nostril” = perception of peanut butter odor from a distance, “Score PEA/EUG” = scores in the odor sorting tasks, “Lat correct assignments overall” score in the lateralization test. Lower values indicate better olfactory performance. The figure has been created using Python version 3.8.12 for Linux (https://www.python.org, accessed on 28 January 2022) and Seaborn Python data visualization library (https://seaborn.pydata.org, accessed on 28 January 2022 [65]).

None of the variables or cases had to be excluded as the 20% cut-off for missing values was not exceeded. Outliers were not detected. After imputation of a total of 79 missing values, the olfaction-related data comprised a 135 × 15 matrix with d = 15 variables recorded from n = 135 patients (Figure 2).

### 3.1. Differences on Olfaction Related Parameters with Respect to the Olfactory Diagnosis

According to the clinical olfactory diagnosis, based on the TDI score of the Sniffin’ Sticks test battery, n = 117 participants (73 women, 44 men) had a normal olfactory function and n = 18 (10 women, eight men) had impaired but partially preserved function (hyposmia). Only the olfactory threshold to PEA, odor discrimination, and identification, which defines the clinical olfactory diagnosis, differed statically significantly when α correction for multiple testing was applied (d = 18 variables including the d = 15 olfaction-related variables in addition to age, sex, and BMI; details not shown). In addition, normosmic subjects performed better in the discrimination task (mean score = 2.35) than hyposmic subjects (mean score = 1.78; Mann–Whitney U = 685.5, *p =* 0.013), had slightly higher values of the errors made in the PEA sorting task when corrected for the time required for this test (0.061631 versus 0.047442; U = 1364.5, *p =* 0.0438), and were younger (mean age = 29.5 years) than the latter (mean age 37 years; U=1424.0, *p =* 0.016). Both did not pass α correction, and the sexes were equally distributed between olfactory diagnoses (χ^2^ = 0.0869, *p* = 0.768).

### 3.2. Variance and Covariance Structure of Olfaction-Related Variables

**Figure 3:**
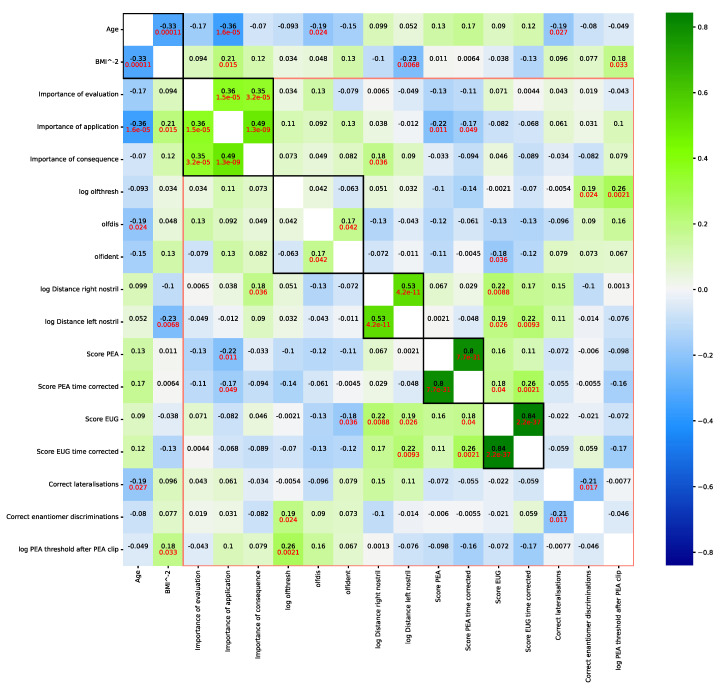
Correlations of olfaction-related data collected from n = 135 subjects, transformed to normal distribution and with missing values imputed. Age and BMI were additionally included. The correlation matrix is color-coded according to the strength and direction of the correlation. Each cell is labeled with the value of Pearson’s r in black numbers. If the correlation is significant, the p-value is indicated in red numbers below the correlation coefficient. The diagonal of the correlations of each variable with itself has been omitted. Variables belonging to the same subtest are highlighted by black rectangles to increase distinction of intra- and intertest correlations. Olfactory variables are outlined in red. Variable names, if not self-explaining: “olfthresh“ = olfactory threshold to phenyl ethyl alcohol (PEA), “olfdis” = score in the odor discrimination task, “olfident” = score in the odor identification task, “log Distance right/left nostril” = perception of peanut butter odor from a distance, “Score PEA/EUG” = scores in the odor sorting tasks, “Lat correct assignments overall” score in the lateralization test. The figure has been created using Python version 3.8.12 for Linux (https://www.python.org, accessed on 28 January 2022) and Seaborn Python data visualization library (https://seaborn.pydata.org, accessed on 28 January 2022 [65]).

Correlations between olfaction-related variables (Figure 3) were statistically significant within the items of the tests of the importance of olfaction and within the items of the distances of the odors to the nostrils measured during the peanut butter test. Subtests of the Sniffin’ Sticks test battery also correlated, excluding the olfactory threshold. This is consistent with the somewhat different position of the olfactory threshold repeatedly observed previously within the three subtests of this test battery [66,67]. The variable “Score EUG” (for variable names, see legend of Table 1) had the largest number of significant correlations with variables recorded in test batteries other than the one to which the particular variable belongs.

**Figure 4:**
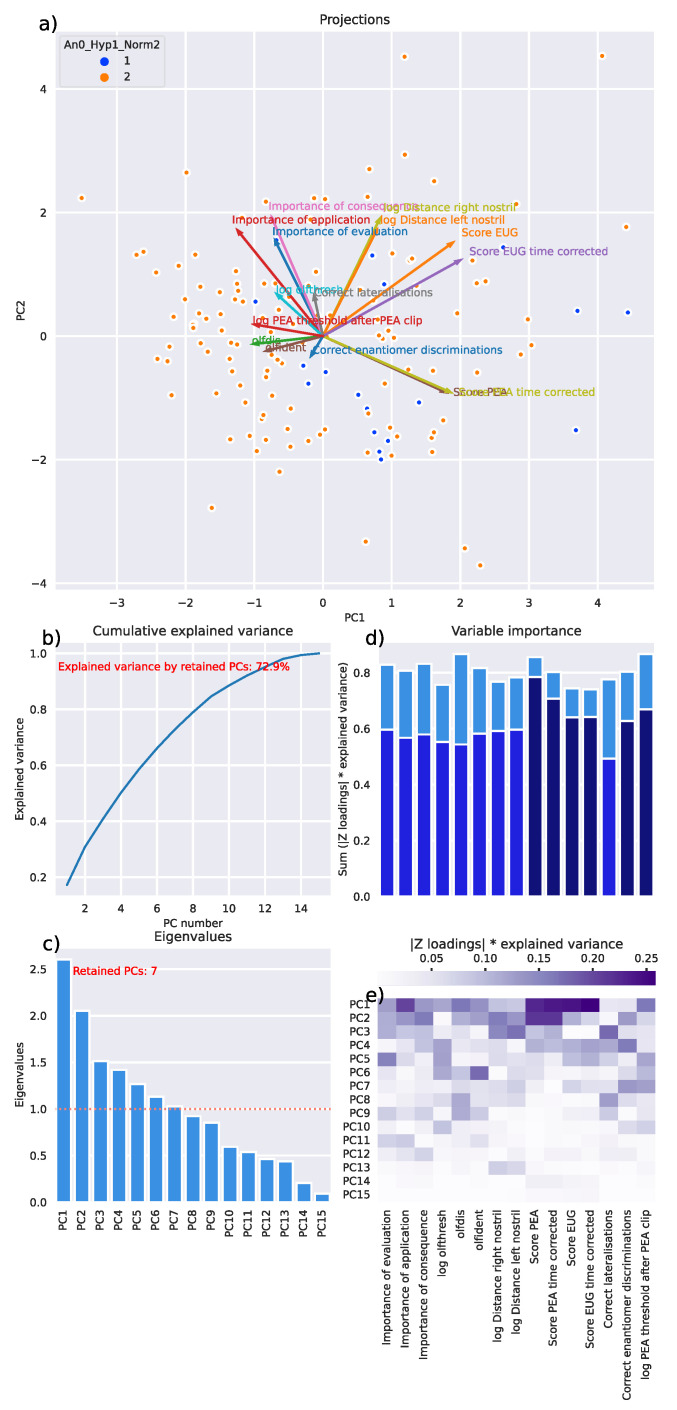
Results of a principal component analysis (PCA)-based projection of centered and standardized olfaction-related data. (**a**) Projection of olfactory data collected in d = 15 olfactory variables from n = 135 subjects onto the first two principal component levels. Data points originating from subjects with normosmia or hyposmia are colored red and blue, respectively. (**b**) Line plot of cumulative explained variance with increasing number of principal components (PCs). (**c**) Bar graph of the eigenvalues of each of the 15 PCs. Seven PCs had an eigenvalue > 1 and were selected for further data analysis such as clustering. The overlaid biplot (red lines) shows the variables as vectors in the PC projection space. (**d**) Contribution of each variable to the principal components, normalized for the contribution of each PC to the explanation of the total variance. The lighter blue bars show the significance of the variables across the entire PC space, while the darker blue bars overlaying them show the contribution when only the relevant 7 PCs are considered. The 6 dark blue bars indicate those selected by item categorization using computed ABC analysis as the most informative variables placed in ABC set “A”. The bar chart shows the column sums of the heat map shown below in panel (**e**) Z-normalized correlation matrix between the original z-transformed dataset and the PC space, normalized by the explained variance. The figure has been created using Python version 3.8.12 for Linux (https://www.python.org, accessed on 28 January 2022) and Seaborn Python data visualization library (https://seaborn.pydata.org, accessed on 28 January 2022 [65]).

PCA yielded d = 7 components with eigenvalues >1, which together explained 72.9% of the total variance of the olfaction-related variables (Figure 4). Variables contributing most to the relevant PCs comprised “Score PEA“, “Score PEA time corrected”, “Score EUG“, “Score EUG time corrected”, “Correct enantiomer discriminations“, and “log PEA threshold after PEA clip“.

### 3.3. Cluster Structure of Olfaction-Related Variables

**Figure 5:**
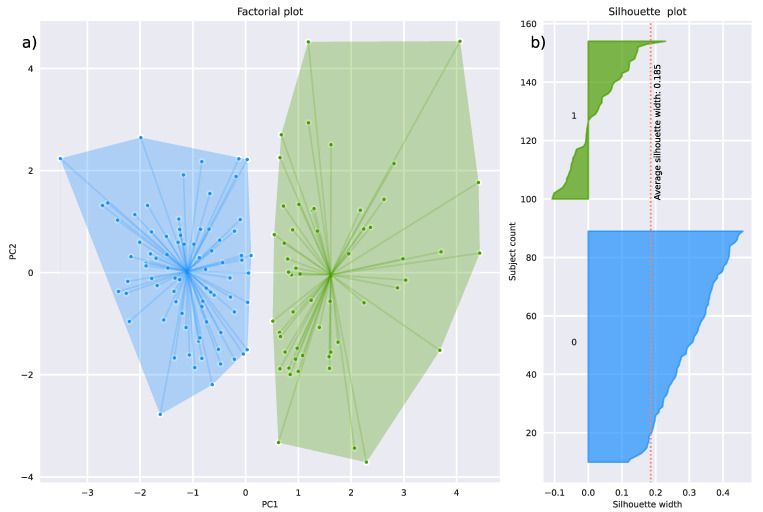
Clustering of the d = 15 olfaction-related parameters. (**a**) Factorial plot of the individual data points on a principal components map, obtained following k-means clustering. The colored areas visualize the cluster separation. The cluster members are connected by straight lines with their respective cluster centers. (**b**) Silhouette plot associated with the cluster solution presented in panel (**a**). The horizontal bars show the average distance of each data point in a cluster is to points in neighboring cluster(s), scaled in the range of [−1, 1] [48]. The figure has been created using Python version 3.8.12 for Linux (https://www.python.org, accessed on 28 January 2022) and Seaborn Python data visualization library (https://seaborn.pydata.org, accessed on 28 January 2022 [65]).

For the olfactory data projected on the seven retained PCs, the calculation of the average silhouette widths of k = 2 to 5 clusters resulted in the best cluster solution with k = 2 k-means clusters (Supplementary Figures S1 and S2). The resulting two clusters, comprising n = 80 (“cluster 0”) and n = 55 (“cluster 1”) subjects (Figure 5), showed a moderate average silhouette width of 0.227 in 20 random resampling runs and an average adjusted Rand index = 0.55.

**Figure 6:**
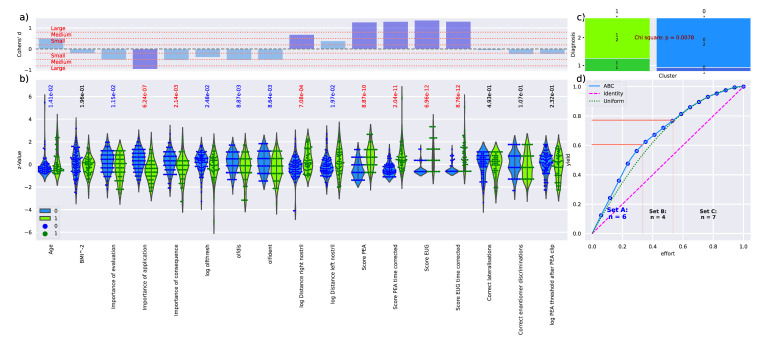
Differences of the d = 15 olfaction-related variables between the two k-means clusters. (**a**) Effect size calculated as Cohen’s d for the parameters used for clustering, and age and the reciprocal square transformed BMI as demographic parameters of known or possible interest of an olfactory context. The effect size of Cohen’s d > 0.2, > 0.5 or > 0.8 generally regarded as small, medium, or large effects are indicated as horizontal dotted lines. Positive values indicate larger values in cluster 1 than in cluster 0. The variables with the most relevant effect sizes according to item categorization (see panel d) are plotted in darker blue color. Variable names, if not self-explaining: “olfthresh“ = olfactory threshold to phenyl ethyl alcohol (PEA), “olfdis” = score in the odor discrimination task, “olfident” = score in the odor identification task. Higher values indicate better olfactory performance. “log Distance right/left nostril” = perception of peanut butter odor from a distance, “Score PEA/EUG” = scores in the odor sorting tasks, “Lat correct assignments overall” score in the lateralization test. Lower values indicate better olfactory performance. (**b**) Z Individual data points, z-transformed to enhance visualization of group differences across different original scales of the values, plotted as dots on violin plots showing the probability density distribution of the variables, and in addition, boxplots provide basic descriptive statistics. Statistical significance of the differences between the clusters for each variable was analyzed by performing Mann–Whitney U-tests. The obtained p-values are shown above the respective variables, with color coding for black = “not significant”, *p* > 0.05), blue = “significant but not passing α correction”, red = “significant with α correction”. (**c**) Mosaic plot of the contingency table of cluster membership (x-axis) versus olfactory diagnosis of hyposmia or normosmia (y-axis). (**d**) ABC analysis plot (blue line) showing the cumulative distribution function of the absolute effect sizes, along with the identity distribution, *x_i_* = constant (magenta line), i.e., each variable has the same effect in terms of inter-cluster differences, and the uniform distribution, i.e., each variable had the same chance to distinguish between cluster (for further details about computed ABC analysis, see [44]). The red lines indicate the borders between ABC subsets “A”, “B”, and “C”. Subset “A” containing d = 6 variables is regarded as containing the most relevant variables for cluster distinction (marked in darker blue in panel a). The figure has been created using Python version 3.8.12 for Linux (https://www.python.org, accessed on 28 January 2022) and Seaborn Python data visualization library (https://seaborn.pydata.org, accessed on 28 January 2022 [65]) and our Python package “ABCanalysis” (https://github.com/JornLotsch/ABCanalysis, accessed on 28 January 2022).

Other than observed with the different olfactory diagnoses (see above), the clusters differed statistically significantly in most olfaction-related parameters (Figure 6). Differences were statistically significant for d = 7 variables when applying a correction; the p-values are shown in Figure 6b. The subtest of the Sniffin’ Sticks test battery failed α correction but provided *p* < 0.05. Subjects assigned to cluster 0 had higher olfactory subtest scores, were slightly younger (mean age 28 ± 9.7 years versus 34 ± 13.2 years in cluster 1) and made fewer errors in the PEA and eugenol sorting tasks. Accordingly, cluster 0 included significantly fewer subjects with hyposmia (Figure 6c). Cluster 0 also contained more women (56 and 24 men, cluster 1: n = 27 women and 28 men, χ^2^ = 5.166, *p =* 0.023). This needs to be regarded in the above-mentioned context that men and women did not differ with respect to the assignment to olfactory diagnostic groups.

**Figure 7:**
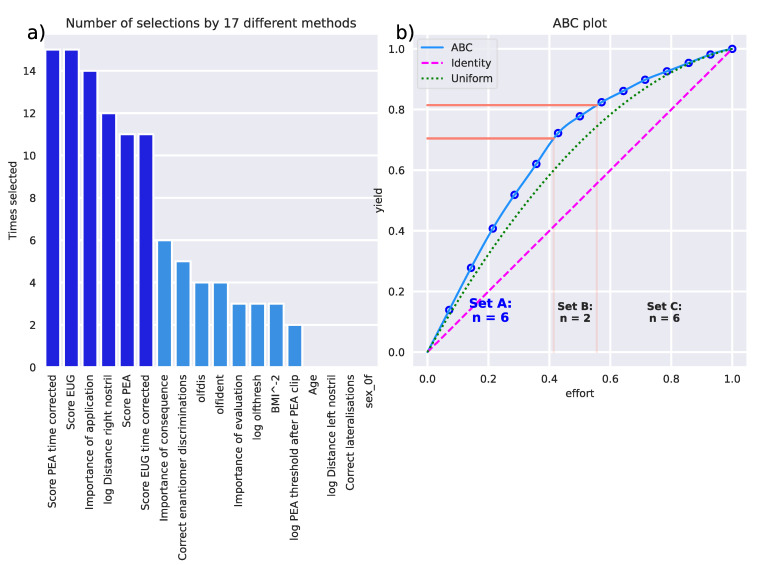
Identification of the variables that were most informative in assigning a subject to the k-means clusters. Feature selection by 17 different methods listed in Table 1. (**a**) The sum score of selections of each variable across the methods was subjected to a computed ABC analysis to identify the most informative variables for all methods of feature selection (row sums in Table 1). The darker blue bars indicate the variables selected for the reduced feature set resulting from ABC analysis-based item categorization. (**b**) ABC analysis plot (blue line) showing the cumulative distribution function of the sums of occurrences in ABC category “A” in the ABC analyses previously performed with each feature selection method separately. The red lines show the boundaries between the ABC subsets “A”, “B” and “C”. Category “A” with d = 6 variables is considered to include the most relevant variables for cluster discrimination (marked in darker blue in panel a). The figure was created using Python version 3.8.12 for Linux (https://www.python.org, accessed on 28 January 2022), with the seaborn statistical data visualization package (https://seaborn.pydata.org, accessed on 28 January 2022 [65]) and our Python package “ABCanalysis” (https://github.com/JornLotsch/ABCanalysis, accessed on 28 January 2022). Variable names, if not self-explaining: “olfthresh“ = olfactory threshold to phenyl ethyl alcohol (PEA), “olfdis” = score in the odor discrimination task, “olfident” = score in the odor identification task, log Distance right/left nostril” = perception of peanut butter odor from a distance, “Score PEA/EUG” = scores in the odor sorting tasks, “Lat correct assignments overall” score in the lateralization test.

**Figure 8:**
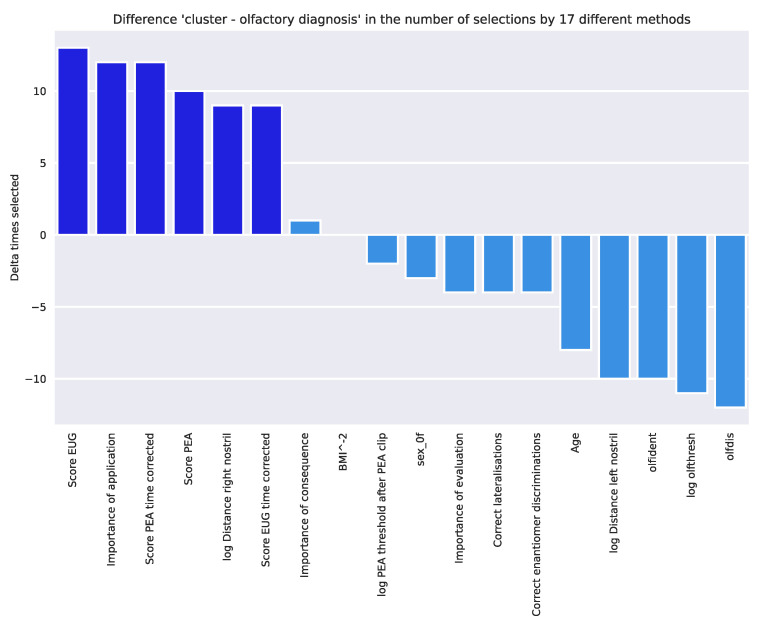
Differences in sum scores of selections of each variable across 17 feature selection methods when the target was the olfaction-related cluster versus the target defined as the olfactory diagnosis of normosmia or hyposmia. The variables selected as informative for the cluster structure are plotted in darker blue. The figure has been created using Python version 3.8.12 for Linux (https://www.python.org, accessed on 28 January 2022) and Seaborn Python data visualization library (https://seaborn.pydata.org, accessed on 28 January 2022 [65]) and our Python package “ABCanalysis” (https://github.com/JornLotsch/ABCanalysis, accessed on 28 January 2022). Variable names, if not self-explaining: “olfthresh“ = olfactory threshold to phenyl ethyl alcohol (PEA), “olfdis” = score in the odor discrimination task, “olfident” = score in the odor identification task, log Distance right/left nostril” = perception of peanut butter odor from a distance, “Score PEA/EUG” = scores in the odor sorting tasks, “Lat correct assignments overall” score in the lateralization test.

The different feature selection techniques (Table 1) pointed to d = 6 variables (Figure 7) as most informative for the detected cluster structure in the olfaction-related data. According to the sum score of the selection by the different techniques, the variables “Score PEA time corrected”, “Score EUG“, “Importance of application“, “log Distance right nostril”, “Score EUG time corrected”, and “Score PEA“ were placed in the ABC category “A”. With these variables, the algorithms could be trained to assign a subject from the initially separated validation dataset to the correct cluster as accurately as with the full feature set (Table 2). Even when using only “Score EUG“ and “Score PEA“, cluster assignment remained similarly good. In contrast, when tuning the algorithms for olfactory diagnosis instead of cluster assignment, class assignment was still possible with the full feature set with median balanced accuracy well above guessing level, while the reduced feature sets failed and did not provide a better cluster assignment than by guessing, as shown by the balanced accuracies close to 50%. However, the small sample of only n = 18 subjects with hyposmia pushed the algorithms to their limits, as suggested by the large confidence intervals of the performance measures, which spanned 50% even with the full feature set. With due caution in interpreting these results, rerunning the feature selection for the olfactory diagnoses and subtracting the obtained sum scores from those obtained when clusters were selected allowed to evaluate the variables according to their relevance to the olfaction-related clusters compared with the olfactory diagnoses, which was fully consistent with the expectations that the components of the olfactory test were most relevant to the diagnosis while highlighting that the clusters provided a different type of olfaction-related subgrouping (Figure 8).

## 4. Discussion

Evaluation of several potential additions to the standard paradigm of odor threshold, discrimination, and identification clinical olfactory testing supported the hypothesis that informative sensory olfactory phenotypes are likely to be more complex. Clusters of subjects that emerged in the pattern formed by various parameters of olfaction did not simply reproduce the grouping by the olfactory diagnosis. Four of the six variables identified as most relevant to the detected cluster structure were the results of sorting tasks of odors at different dilutions. Thus, the accepted grouping of olfactory function into normosmic subjects or subjects with impaired function, based on the three accepted sensory dimensions of olfaction, seems to be modified by the addition of a simple further sensory olfactory task.

The sorting tasks for ascending dilutions of PEA and eugenol would provide a well-fitting extension of the clinical test of olfactory function, assessing olfactory threshold, odor discrimination, and odor identification, by maintaining the sensory focus of the test battery while providing additional phenotypic information. The sorting task requires numerous olfactory skills, including the ability to discriminate odors, memorize olfactory information, and separate odors of various intensities. The closely related time-corrected performances were highly correlated with the latter but have the disadvantage that they do not penalize premature termination of the test. Therefore, a preference of the time-corrected variables to raw errors in the sorting regardless of the time required would have needed further modification of these variables or a redesign of the present sorting task with the setting of a time limit. In contrast, the items assessing the importance of the sense of smell to the person’s life, scored as “Importance of application“, which was also among highly ranked variables, would add another facet of the sense of smell to the test battery that would interfere with its focus of the sensory function of smell.

The present approach to feature selection for cluster explanation followed the standard workflow of classifier creation in machine learning, including sample splits into training/test/validation, cross-validation, and final performance testing. Supervised analyses used for cluster explanation were implemented with different methods to avoid the results reflecting certain properties of a single feature selection method. The final goal of supervised machine learning was not the creation of a well-performing classifier suitable as a diagnostic tool, but to explain the structure in the data to provide a description that provides better knowledge about the dataset. This knowledge-discovery approach assumes that if a classifier can be trained to recognize a subject’s membership in a subgroup or cluster better than by guessing, the features, i.e., the olfactory relevant variables in the data set at hand plus age, sex, and BMI, that the classifier needs to perform this task will contain relevant information about the cluster structure being addressed.

The present subgroup structure in relation to the classical olfactory diagnoses was unbalanced with a significantly larger proportion of subjects with normal olfactory function. This was considered when exploring which variables were informative for the detected cluster structure but less for the subgrouping for olfactory diagnosis. However, the present cohort was closer to a random sample that was considered more appropriate for studying relevant phenotypes related to olfaction beyond the classic clinical odor diagnoses than a balanced cohort containing all odor diagnosis groups in a balanced proportion, which would have required a strong enrichment of the cohort with subjects with impaired olfactory function, who are underrepresented in the general population. Specifically, up to 15% of the general population exhibit olfactory loss with an additional 3–5% showing functional anosmia [68,69]. The composition of the present cohort of n = 117 subjects with normal olfactory function and 18 subjects with impaired function did not differ significantly from the composition of the general population of 82 and 18 persons, respectively (χ^2^ = 0.964, *p =* 0.3261). In addition, clusters based on olfaction-related information could be found in a group where most subjects had normal olfactory function, which rather reinforces the present results of a more complex olfactory phenotype than reflected by the three subtests of a standard clinical test of the sense of smell.

## 5. Conclusions

The group structures that emerge from olfactory test data in subjects with normal or impaired sense of smell are more complex than the three accepted sensory dimensions of smell. A cluster structure resulting from various different tests of olfactory function could not be explained by the results of the three subjects of a standard clinical test alone. Moreover, the grouping of olfactory function into normosmic subjects or subjects with impaired function, based on the three accepted sensory dimensions of olfaction, was disrupted by the addition of a simple further sensory olfactory task. The present analysis ended with the proposal of a specific addition to the sensory olfactory test battery established in clinical practice. Expanding the olfactory test battery to include the two proposed odor dilutions sorting tasks now requires a prospective evaluation using larger sample sizes and systematically including the classic olfactory diagnoses, i.e., the inclusion of a balanced sample in terms of clinical olfactory diagnoses allowing to capture both hyposmia and anosmia, as well as normal olfactory function, which was prevalent in the present cohort. In addition, any expansion of an established olfactory test would need to address the specifics of including additional scores, such as the direction of the measurements or their final scaling, to fit within the so-far consistent 0 or 1 to 16 scaling of the current Sniffin’ Sticks test battery.

## Figures and Tables

**Table 1:** Identification of the variables that were most informative in assigning a subject to the k-means clusters. Features were selected according to importance of the variables in the PCA projection of the olfaction-related data, using univariate methods implemented as effect sizes calculated as Cohen’s d (“C’d”) and the “SelectKBest” (SKB) method with model-based grid search to determine how many variables k to be selected, and model-based methods implemented as “SelectFromModel“ (SFM), recursive feature elimination (RFE), and forward and backward sequential feature selection (SFS) used with linear support vector machines (SVM), random forests (RF), and logistic regression (LogReg), in a 100-fold cross-validation scenario run on the initially drawn training data subset. All the methods provided a ranking of the variables for their relevance to the cluster structure, which was subjected to computed ABC analyses, and category “A” containing the most important items was retained. The sum of occurrences in category “A” is shown in the third column from the left. The selection for the final dataset is indicated in the next column to the right of the variable name (“X”). Note that age, sex, and BMI were not used in the PCA projection and therefore could not be selected as relevant features in the PCA. Variable names, if not self-explaining: “olfthresh“ = olfactory threshold to phenyl ethyl alcohol (PEA), “olfdis” = score in the odor discrimination task, “olfident” = score in the odor identification task, “log Distance right/left nostril” = perception of peanut butter odor from a distance, “Score PEA/EUG” = scores in the odor sorting tasks, “Lat correct assignments overall” score in the lateralization test.

Variable	Final	Sum	PCA	Univariate Feature Selection				Model Based Feature Selection											
				C’ d	SKB			SFM			RFE			SFS forward			SFS backward		
					SVM	RF	LogReg	SVM	RF	LogReg	SVM	RF	LogReg	SVM	RF	LogReg	SVM	RF	LogReg
**Age**																			
**BMI^−2^**		3												1	1	1			
**Importance of evaluation**		3									1		1				1		
**Importance of application**	X	14		1	1	1	1		1		1	1	1	1	1	1	1	1	1
**Importance of consequence**		6			1	1	1							1	1			1	
**log olfthresh**		3			1		1					1							
**olfdis**		4			1		1										1		1
**olfident**		4									1		1				1		1
**log Distance right nostril**	X	12			1	1	1				1	1	1	1	1	1	1	1	1
**log Distance left nostril**																			
**Score PEA**	X	11	1	1	1	1	1				1	1	1	1	1			1	
**Score PEA time corrected**	X	15	1	1	1	1	1		1		1	1	1	1	1	1	1	1	1
**Score EUG**	X	15	1	1	1	1	1		1		1	1	1	1	1	1	1	1	1
**Score EUG time corrected**	X	11	1	1	1	1	1		1		1	1	1					1	1
**Lat correct assignments overall**																			
**Correct enantiomer discriminations**		5	1											1	1	1		1	
**log PEA threshold after PEA clip**		2	1														1		
**Sex**																			

**Table 2:** Validation of the feature sets by training three different classifiers (linear support vector machine, SVM, random forests, and logistic regression) with subsets of the training dataset with all variables (d = 15 olfaction-related features and age, sex, and BMI) as “full” feature set and with the d = 6 variables that had resulted from the feature selection steps shown in Table 1 as “reduced” feature set. In addition, the classification task was repeated using only the variables “Score PEA“ and “Score EUG“ to test the hypothesis that these provide an informative addition to the olfactory test. The classification task was designed for the cluster assignment. In addition, after re-tuning the algorithms, the task was repeated with the olfactory diagnosis as target. Shown are the medians and nonparametric 95% confidence intervals (2.5th to 97.5th percentiles) from 100-fold cross-validation runs.

Classifier	Performance Measure	Olfaction-Related Clusters			Olfactory Diagnoses		
		Feature set			Feature set		
		Full	Reduced	Sparse	Full	Reduced	Sparse
**SVM**	Balanced accuracy	0.96 (0.91–1)	1 (0.91–1)	0.88 (0.82–0.94)	0.77 (0.46–1)	0.5 (0.47–0.5)	0.5 (0.5–0.5)
**Random forests**		0.87 (0.77–0.98)	0.86 (0.78–0.96)	0.77 (0.72–0.9)	0.63 (0.5–0.75)	0.5 (0.47–0.5)	0.47 (0.47–0.5)
**Logistic regression**		1 (0.95–1)	1 (1–1)	0.88 (0.82–0.94)	0.75 (0.5–0.88)	0.5 (0.47–0.5)	0.5 (0.5–0.5)

## Data Availability

Data available on request from the senior author. Parts of the Python code created for data analysis are available at https://github.com/JornLotsch/OdorSortingReport (accessed on 28 January 2022). **Acknowldedgment:** We would like to thank aspUraclip, Berlin-Schönefeld, Germany, for providing the nasal clips. We also would like to thank Yling Mai, shown in Figure 1, who consented orally and in writing to the publication of the photographs within the context of this publication.

## Supplementary Materials

The following supporting information can be downloaded at: https://www.mdpi.com/article/10.3390/jcm11144012/s1, “SupplementaryFigures.docx” (Microsoft Office Open XML format as a Microsoft Word® document), which includes silhouette plots with [2, …, 5] k-means and hierarchical clusters. Figure S1. k-means based clusters, using the Euclidean distance between the retained principal components. The figure has been created using Python version 3.8.12 for Linux (https://www.python.org, accessed on 28 January 2022) and Seaborn Python data visualization library (https://seaborn.pydata.org, accessed on 28 January 2022). Figure S2. Hierarchical (Ward) based clusters using the Euclidean distance between the retained principal compo-nents. The figure has been created using Python version 3.8.12 for Linux (https://www.python.org, accessed on 28 January 2022) and Seaborn Python data visualization library (https://seaborn.pydata.org, accessed on 28 January 2022).
