# Supplementary material for: Sorting of Odor Dilutions Is a Meaningful Addition to Assessments of Olfactory Function as Suggested by Machine-Learning-Based Analyses
Source: J Clin Med. 2022 Jul 11;11(14):4012. doi: 10.3390/jcm11144012 (PMC9317381; doi:10.3390/jcm11144012)

Supplemental figures: Silhouette plots of k-means and hierarchical clustering with k = [2,…5] clusters

Supplemental Figure S1: k-means based clusters, using the Euclidean distance between the retained principal components. The figure has been created using Python version 3.8.12 for Linux (https://www.python.org) and Seaborn Python data visualization library (https://seaborn.pydata.org).

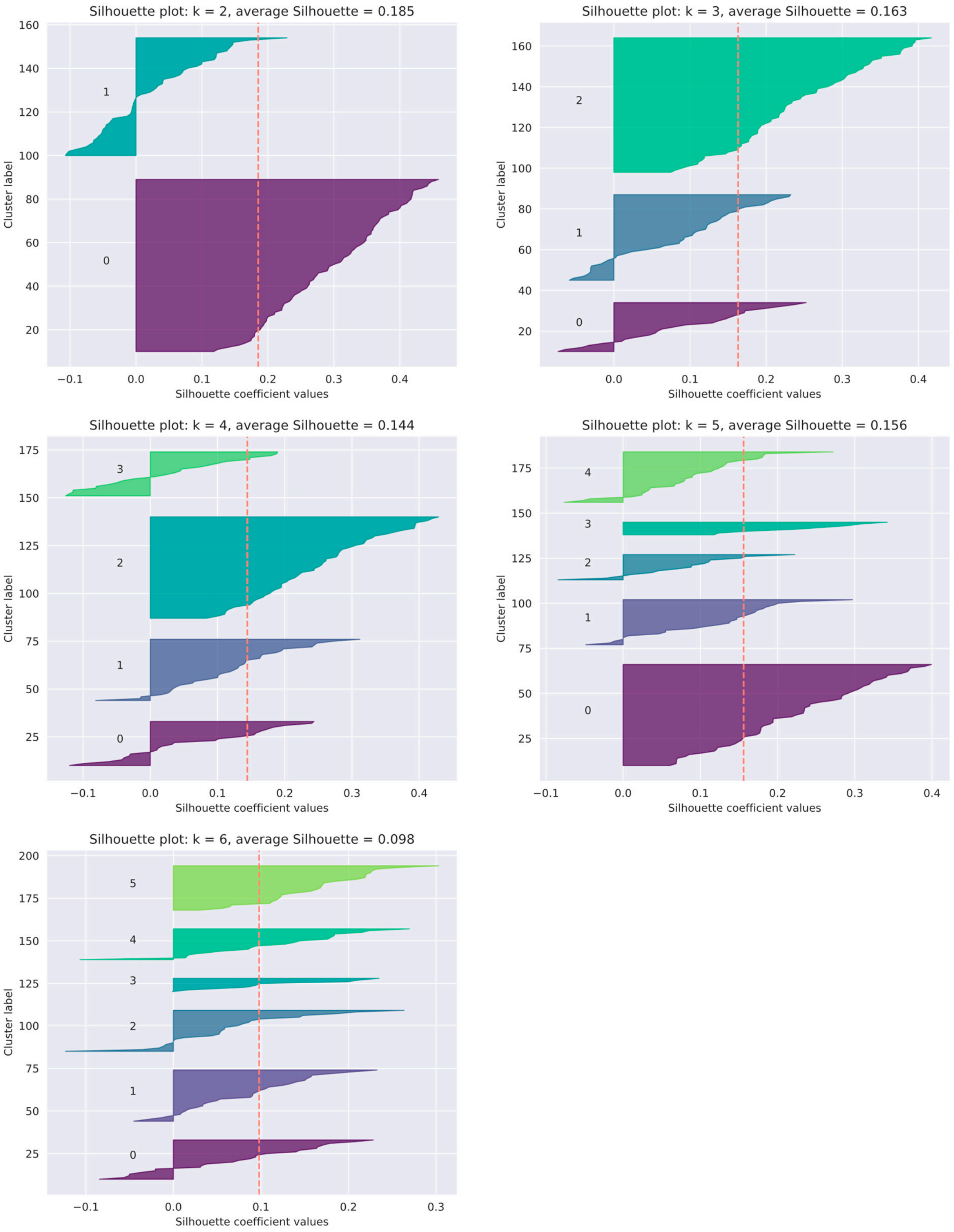

Supplemental Figure S2: Hierarchical (Ward) based clusters using the Euclidean distance between the retained principal components. The figure has been created using Python version 3.8.12 for Linux (https://www.python.org) and Seaborn Python data visualization library (https://seaborn.pydata.org).

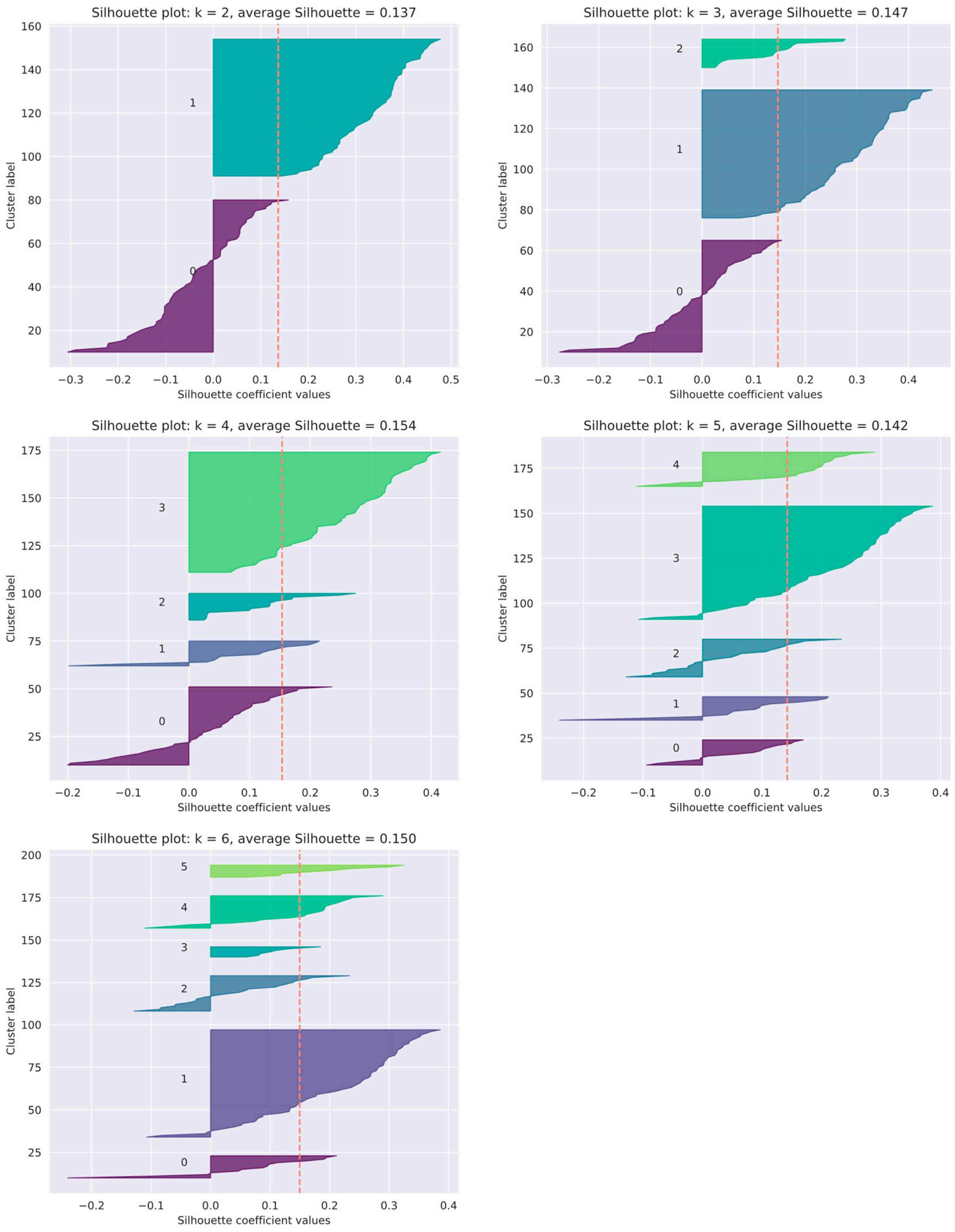

Supplement: Supplementary file 1 [file jcm-11-04012-s001.zip › jcm-1744142-supplementary.pdf]
